# Persistent Anti-*Borrelia* IgM Antibodies without Lyme Borreliosis in the Clinical and Immunological Context

**DOI:** 10.1128/Spectrum.01020-21

**Published:** 2021-12-22

**Authors:** Mateusz Markowicz, Michael Reiter, Jutta Gamper, Gerold Stanek, Hannes Stockinger

**Affiliations:** a Institute for Hygiene and Applied Immunology, Center for Pathophysiology, Infectiology and Immunology, Medical University of Vienna, Vienna, Austria; b Center for Medical Statistics, Informatics and Intelligent Systems, Section for Medical Statistics, Medical University of Vienna, Vienna, Austria; Memorial Sloan Kettering Cancer Center

**Keywords:** *Borrelia burgdorferi*, Lyme disease, OspC, persistent IgM, cross-reactivity, Lyme borreliosis

## Abstract

The aim of the study was to investigate the etiology of persistent IgM antibodies against Borrelia burgdorferi sensu lato (sl) and to analyze their association with nonspecific symptoms. The study group comprised individuals with persistent IgM antibodies in the absence of IgG. The relation between ELISA values and time elapsed since past erythema migrans (EM) was analyzed. Previous antibiotic treatments were assessed. The association between persistent IgM and nonspecific symptoms was evaluated statistically. Specificity of IgM antibodies for outer surface protein C (OspC) of B. burgdorferi sl was examined by immunoblotting. Further, we investigated the cross-reactivity with *Borrelia*-unrelated proteins. Fifty-nine patients (46 women; 78%) were included in the study group. The mean IgM-ELISA values did not change significantly during follow-up (median 6.2 months). The mean ELISA value in the study group was dependent on time elapsed since past EM. Nonspecific symptoms improved significantly more often in patients with lower IgM ELISA results. Persistent IgM antibodies were specific for the C-terminal PKKP motif of OspC. Cross-reacting C-terminal PKKP antigens from both human and prokaryotic origins were identified. We demonstrate that the C-terminal PKKP motif plays a main role for the reactivity of persistent Borrelia IgM toward OspC. However, cross-reactivity to other eukaryotic and/or prokaryotic antigens may hamper the specificity of OspC in the serological diagnosis of Lyme borreliosis. Lack of improvement of nonspecific symptoms was associated with higher IgM ELISA values.

**IMPORTANCE** The reactivity of human IgM with the outer surface protein C (OspC) of Borrelia burgdorferi sensu lato is frequently used to detect *Borrelia* specific IgM in commercial immunoassays, and such antibodies usually occur in the early phase of the infection. We identified a group of individuals with persistent *Borrelia* IgM without symptoms of Lyme borreliosis. We used their sera to demonstrate that the C-terminal epitope of OspC binds the IgM. Strikingly, we found that the same epitope occurs also in certain proteins of human and environmental origin; the latter include other bacteria and food plants. Our experimental data show that these *Borrelia*-unrelated proteins cross-react with the OpsC-specific IgM. This knowledge is important for the development of serologic assays for Lyme borreliosis and provides a cross-reactive explanation for the persistence of *Borrelia*-IgM.

## INTRODUCTION

Diagnosis of Lyme borreliosis (LB) is based on clinical manifestation and supportive laboratory test results in the event of disseminated and late manifestations (1, 2). Detection of antibodies against immunogenic and specific antigens of Borrelia burgdorferi sensu lato (sl) is a two-step process comprising a screening test and a supplemental test (3). B. burgdorferi sl induces a strong, highly specific immune response (4). However, in the early phase of infection, such as in patients with erythema migrans (EM), antibodies can be detected only in some individuals. Therefore, the diagnosis is based on clinical characteristics. The infection progresses without antibiotic treatment or in the case of treatment delay. Seroconversion to anti-*Borrelia* IgG occurs after several weeks. However, both IgM and IgG can persist in healthy persons after tick exposure or after treatment for the manifest infection.

The interpretation of serological test results is difficult in the context of nonspecific complaints (5). Persistent IgM without IgG has been explained as resulting from cross-reactions with other antigens or polyclonal stimulation of B cells (6, 7). Although there is no evidence of ongoing infection in such patients, many of them are repeatedly treated with antibiotics. The phenomenon of persistent IgM to *Borrelia* commonly appears in Europe and North America (8–11). Therefore, its etiology and its impact on the occurrence of nonspecific symptoms and on antibiotic treatment deserve detailed investigation.

The aims of the study were (i) to identify the antigens which are reactive in patients with persistent anti-*Borrelia* IgM and in EM patients; (ii) to study cross-reactions to *Borrelia*-unrelated antigens; (iii) to examine whether the number and the improvement of reported symptoms are associated with enzyme-linked immunosorbent assay (ELISA) values; and (iv) to determine the frequency of antibiotic prescriptions in patients with persistent anti-*Borrelia* IgM.

## RESULTS

### Investigation of persistent IgM.

**FIG 1:**
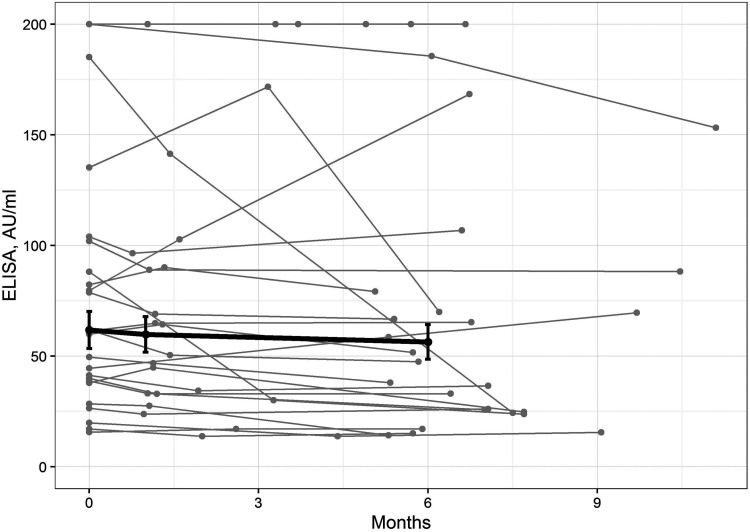
Change in IgM ELISA values over time. Bold line: mean values in study participants with 6 months’ follow-up (error bars indicate standard errors); gray lines: individual ELISA profiles. The points and lines at the maximum ELISA value of 200 AU/ml correspond to more than one patient.

Figure 1 shows the levels of the IgM ELISA values during the median follow-up period of 6.2 months (range 5–11 months). At the first time point, all 59 sera were positive for OspC (100%), 43 (73%) for p41, three (5%) for p39, and two (3%) for variable major protein-like sequence expression (VlsE). Likewise, all sera from the EM controls were reactive to OspC. Reactivity to p41 was found in 13 out of 14 samples (93%). Table 1 shows the differences of the immunoblotting (IB) intensities and ELISA values between the study patients and EM patients.

**TABLE 1:** Comparison of IgM immunoblot band intensities and ELISA values (AU/ml) in study patients and patients with current EM; mean ± standard deviation per group and permutation-adjusted *P values*^*a*^

Antigen	Study (*n* = 59)	EM (*n* = 14)	*P* value (adjusted)
OspCBsp	89.5 ± 38.8	61.29 ± 33.5	**0.0446** ^*b*^
OspCBg	88.36 ± 44.7	61 ± 36.5	0.0767
OspCBb	95.36 ± 48.5	70.59 ± 33.9	0.0899
OspCBa	100 ± 49.1	72.36 ± 33.5	0.0821
p41	56.71 ± 34.2	24.71 ± 23.1	**0.0215**
ELISA	137.19 ± 70.3	71.79 ± 56	**0.0215**

a Values for VlsE and p39 not shown, due to low number of specimens with a positive signal (2 and 3 participants were positive, respectively, in the study group, none in the EM group) OspC, outer surface protein C; Bsp, Borrelia spielmanii; Bg, Borrelia garinii; Bb, Borrelia burgdorferi sensu stricto; Ba, Borrelia afzelii.

b Bold *P* values statistically significant (*P* < 0.05).

IgM antibodies to *Epstein Barr Virus* (EBV), *Herpes simplex virus 1 and 2* (HSV), and *cytomegalovirus* (CMV) were detected in four (7%), in two (4%), and in one participants (2%), respectively. All sera were negative for *Parvovirus B19* (P19V) IgM. The samples were also tested for IgG antibodies against viruses, with 41 (69%) being positive for HSV1, 35 (59%) for CMV, 31 (53%) for P19V, and 13 (22%) for HSV2. IgG to EBV was detected in all samples.

**FIG 2:**
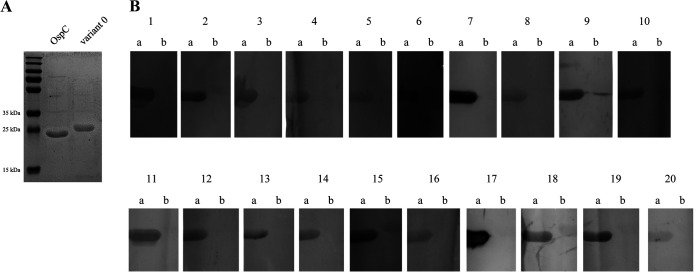
(A) Coomassie Brilliant blue-stained polyacrylamide gel of the purified recombinant OspC proteins. The truncated version of the protein, lacking the 20 C-terminal amino acids (variant 0), is slightly larger as it misses the native stop-codon present in OspC. (B). Immunoblot of patient sera (1-20) against (a) full-length OspC and (b) variant 0 lacking the previously identified 10 amino acid-long IgM epitope.

**FIG 3:**
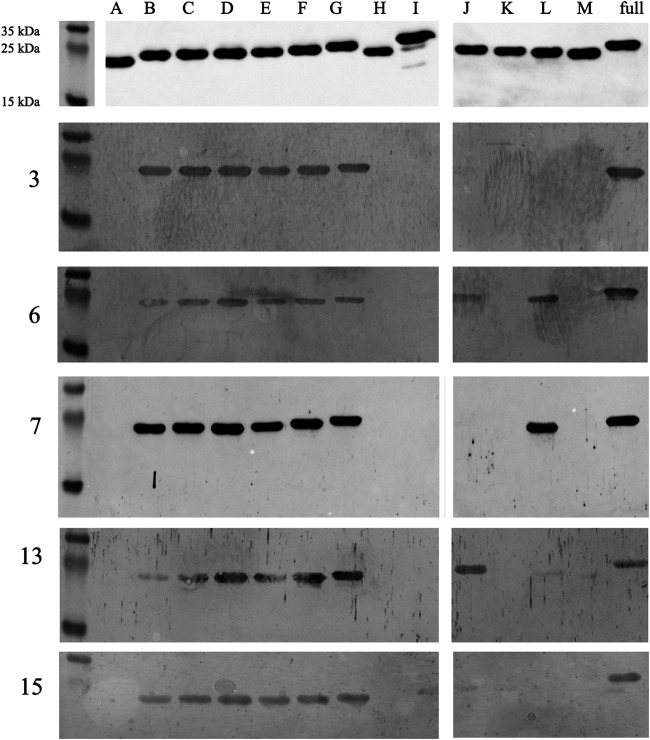
Immunoblots of representative patient sera with the different variants of OspC. Top panel: variants of recombinant OspC (A-M) and full-length OspC probed with an anti His-tag antibody. Lower panels: serum samples (3, 6, 7, 13, 15) from patients with persistent anti-*Borrelia* IgM representing the different staining patterns observed.

The sera from the first 20 participants were used to test the specificity of the IgM antibodies toward a previously identified epitope of OspC comprising the final 10 C-terminal amino acids (12). Using full-length recombinant OspC of B. afzelii strain Pko and a version lacking the 20 C-terminal amino acids (variant 0), we ascertained by IB that all tested sera reacted with the full-length OspC but not with the deletion mutant (Fig. 2 and Table 2). To further narrow down the epitope, we generated more variants of OspC (B-M) by altering the C-terminal sequence (Table 3). Because of a shortage of sample material, we were able to test only 15 of the previously used 20 sera with these variants. All 15 sera tested were specific for the C-terminal “PKKP” epitope, as lack of this sequence (variant H, Table 2) caused nonreactivity (Fig. 3). The stepwise deletion of amino acids 203–209 (variants B–G), that is, the six amino acid upstream of PKKP, had no influence on the binding of any sera, indicating that the very C-terminal PKKP motif is the minimal essential epitope of all OspC-reactive persistent IgM antibodies. Removing the proline residue of the PKKP tetramer from either the N- or C-terminus (variants J and L, respectively) abrogated the binding of nine sera but kept it for five (sera 5, 6, 9, 12, 13). Three of the latter (sera 9, 12, 13) also reacted with variant M, narrowing down their epitope to KK and KP, respectively. One sample, serum 7, reacted with variant L but with no other PKKP variant considering KKP as its epitope. When we deleted the complete decamer “PVVAESPKKP” from the C-terminus and inserted it in the middle of the protein (at amino acid position 122–variant I), only one serum sample (15) was weakly yet clearly reactive (Table 2, Fig. 3). In summary, all persistent anti-*Borrelia* OspC IgM antibodies recognize epitope PKKP, but only when located at the very C-terminus (with the exception of serum 15). This finding allowed us to investigate the possibility of cross-reactions.

**TABLE 2:** Specificity of IgM antibodies for OspC variants^*a*^

Serum ID	A	B	C	D	E	F	G	H	I	J	K	L	M	OspC
1	−	+	+	+	+	+	+	−	−	−	−	−	n.d.	+
2	−	+	+	+	+	+	+	−	n.d.	n.d.	n.d.	n.d.	n.d.	+
3	−	+	+	+	+	+	+	−	−	−	−	−	−	+
4	−	+	+	+	+	+	+	−	−	−	−	−	n.d.	+
5	−	+	+	+	+	+	+	−	−	+	−	+/−	−	+
6	−	+	+	+	+	+	+	−	−	+	−	+	−	+
7	−	+	+	+	+	+	+	−	−	−	−	+	−	+
8	−	+	+	+	+	+	+	−	−	−	−	−	−	+
9	−	+	+	+	+	+	+	−	−	+/−	−	+	+/−	+
10	−	+/−	+/−	+	+	+	+	−	−	−	−	−	n.d.	+
11	−	+	+	+	+	+	+	−	−	−	−	−	n.d.	+
12	−	+	+	+	+	+	+	−	−	+/−	−	+	+/−	+
13	−	+	+	+	+	+	+	−	−	+	−	+/−	+/−	+
14	−	+	+	+	+	+	+	−	−	−	−	−	+/−	+
15	−	+	+	+	+	+	+	−	+	+	+	−	−	+

*^a^*(−) no reaction; (+) reaction; (+/−) weak reaction; n.d., not determined.

**TABLE 3:** OspC variants 0, and A-M created from the full-length protein (top row)^*a*^

Variant	C-terminal OspC peptide sequence of B. afzelii PKo starting at aa position 192^*b*^	Position^*b*^ of in/del
OspC	A L T N S V K E L T S P V V A E S P K K P	
0	A	del. aa 193-212
A	A L T N S V K E L T S	del. aa 203-212
B	A L T N S V K E L T S P K K P	del. aa 203-208
C	A L T N S V K E L T S S P K K P	del. aa 203-207
D	A L T N S V K E L T S E S P K K P	del. aa 203-206
E	A L T N S V K E L T S A E S P K K P	del. aa 203-205
F	A L T N S V K E L T S V A E S P K K P	del. aa 203-204
G	A L T N S V K E L T S V V A E S P K K P	del. aa 203
H	A L T N S V K E L T S P V V A E S	del. aa 209-212
I	C-terminal deletion of PVVAESPKKP and insertion at position 122 within the protein	in. aa 122-131; del. aa 203-212
J	V K E L T S P V V A E S P K K	del. aa 212
K	A L T N S V K E L T S P V V A E S P K	del. aa 211-212
L	A L T N S V K E L T S K K P	del. aa 203-209
M	A L T N S V K E L T S K P	del. aa 203-210

*^a^*aa, amino acid; in, insertion; del, deletion.

*^b^*Position of in/del in amino acid sequence according to NCBI reference Sequence WP_011600714.1.

**FIG 4:**
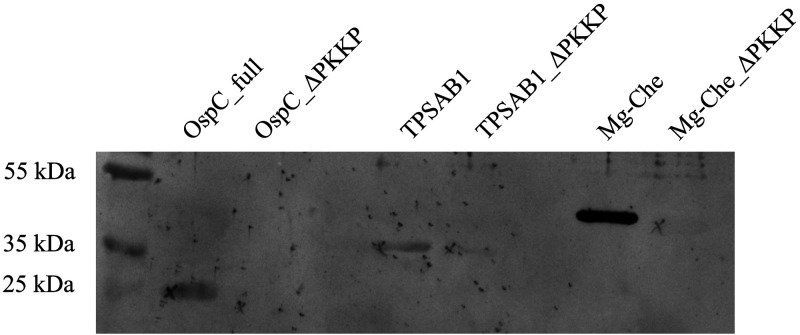
Representative immunoblot of patient serum 12 showing cross-reactivity toward antigens featuring a C-terminal “PKKP” and mutated versions thereof missing this motif (“ΔPKKP”). OspC_full, full-length B. afzelii OspC; TPSAB1, human tryptase 1; Mg-Che, P. aeruginosa Mg-chelatase.

A peptide BLAST search for the IgM epitope comprising the 10 C-terminal amino acids of OspC (PVVAESPKKP) showed that this sequence is specific for Lyme *Borrelia.* However, when we applied the same search algorithm for the C-terminal “PKKP” tetramer, the number of non-*Borrelia* proteins increased to 2638 (2818 database entries, Table S3 in the supplemental material). From these hits, we selected two proteins, one of human origin (TPSAB1) and another of bacterial origin (*bchI*), for subsequent binding assays. We recombinantly expressed these two proteins in E. coli either as a full-length or a PKKP-deleted form and tested them for reactivity with three individual sera. Similar to our findings with OspC, the sera from patients with persistent anti-*Borrelia* IgM reacted to these antigens but lost reactivity when the C-terminal “PKKP” motif was deleted (representative sample, see Fig. 4). Only one sample showed weak reactivity with the mutated version of PKKP-deleted TPSAB1.

### History of EM and presence of IgM antibodies.

**FIG 5:**
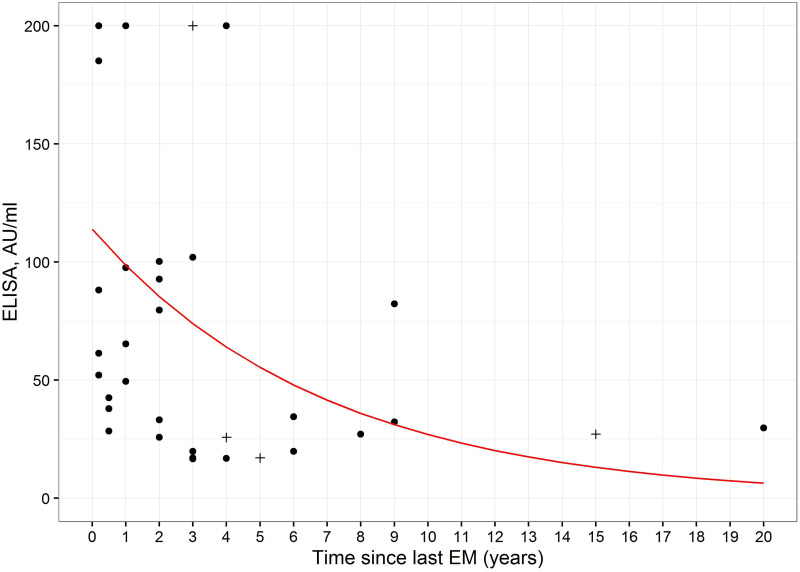
IgM ELISA values as a function of time since previous EM. The red line represents the exponential regression fit. Points marked with + are ELISA values of patients with two EMs in the past. These points were not included to fit the model.

Thirty-two of the 59 patients reported a history of physician-determined EM and four of them had had two such episodes. The nonlinear relation between IgM ELISA values at the first visit and the time since last EM is shown in Figure 5.

**FIG 6:**
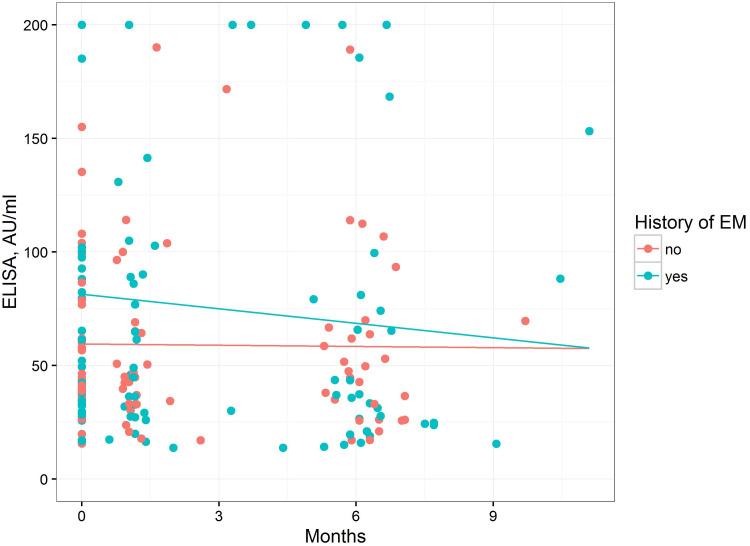
IgM ELISA values over time in patients with or without a history of EM. Regression lines illustrate the results of the linear mixed model.

The differences in IgM ELISA values between patients with and without a history of EM were analyzed as shown in Figure 6. The dynamics of the mean ELISA over time were almost constant in the patients without a history of EM. In contrast, in patients with a history of EM, the ELISA values decreased over time, although the difference was not significant (significant interaction term history:time, *P = *0.0406).

### IgM and nonspecific symptoms.

In total, 45 study participants reported nonspecific symptoms and the mean number of symptoms was 2.4 (range 0–7). Thirty-one participants reported joint pain, 5 joint swellings, 4 back pain, 31 muscle pain, 22 fatigue, 8 forgetfulness, 16 headache, 3 visual problems, and 11 had vertigo. Fourteen study participants reported no complaints. Table 4 shows the mean and standard deviation of initial IgM ELISA according to the number of symptoms. Our analysis of variance (ANOVA) for the 45 participants with symptoms showed no overall association between the ELISA value and number of symptoms (global *F* test, *P = *0.76).

**TABLE 4:** Numbers of patients with the respective number of nonspecific symptoms; mean and standard deviation (SD) of the initial IgM ELISA values

No. of symptoms	0	1	2	3	4	5	6	7
No. of patients	14	6	13	9	7	5	4	1^*a*^
Mean ELISA (AU/ml)	47.7	76.6	68.5	96.5	79.8	105.9	61	17
SD	44.3	63.6	58	62.9	60	65.4	24.2	NA

*^a^*Only one patient showed seven symptoms, the mean is therefore the ELISA value in this patient (SD cannot be computed).

**FIG 7:**
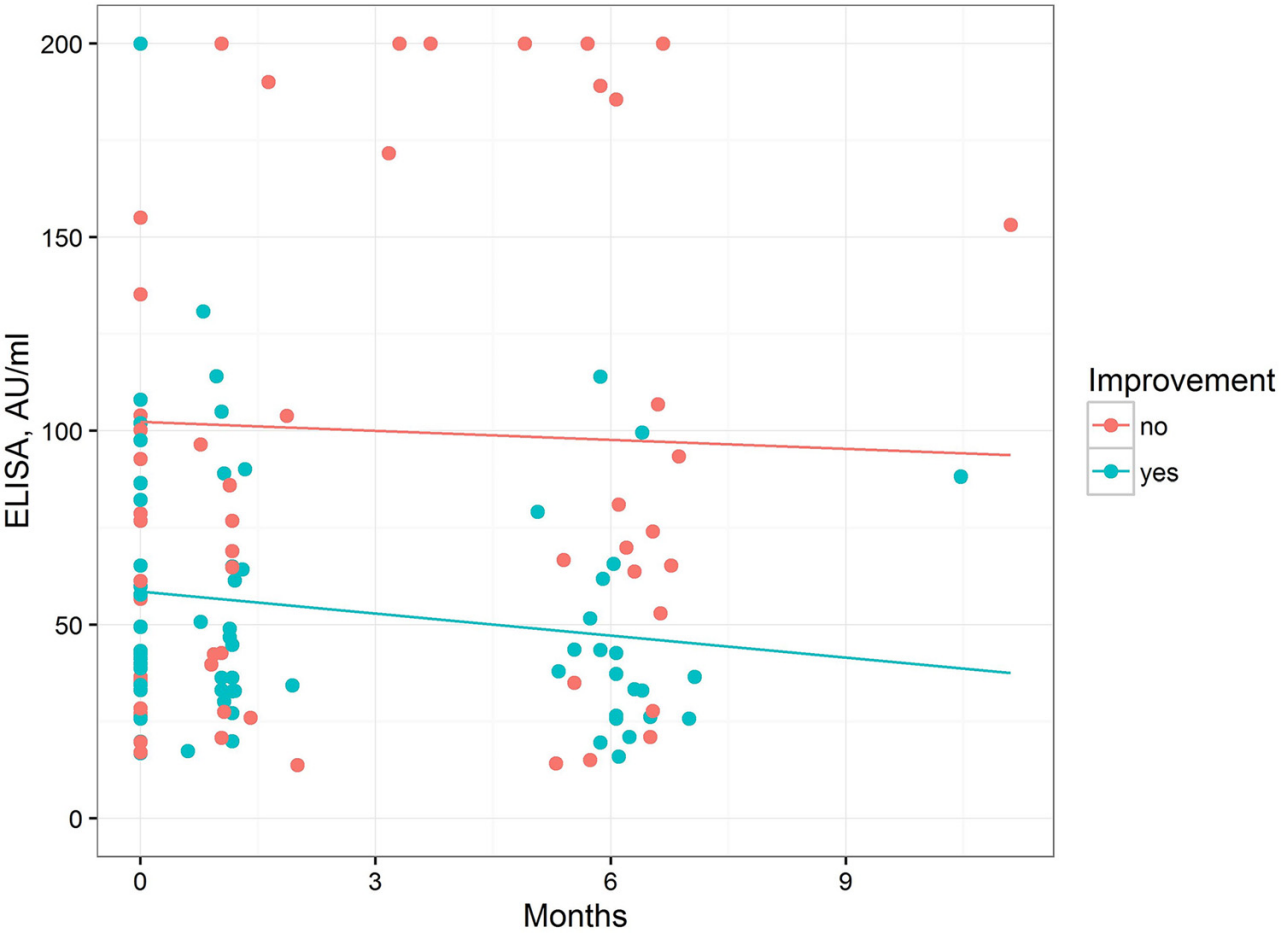
Initial and 6-month IgM ELISA values in 42 symptomatic patients. Regression lines illustrate the results of the linear mixed model.

At the 6-month follow-up, 20 participants reported no improvement, 22 had experienced an improvement, and the improvement was unknown in three. The mean IgM ELISA values were compared using a linear mixed model. The mean values in patients with symptom improvement were significantly lower than those in patients without improvement (*P = *0.0136) (Fig. 7).

### IgM and antibiotic treatment.

In total, 27 (46%) study participants had been treated with antibiotics because of persistent IgM before study inclusion. The treatment had been repeated in some participants because the IgM antibody levels had not decreased. Six participants had been treated twice and three had been prescribed a total of three treatment courses.

## DISCUSSION

The *Borrelia* species that cause Lyme borreliosis (LB) in Europe differ from those in the United States, and this affects serological diagnosis (13). Guidelines in the United States recommend reactivity to at least two different antigens for a positive IB result. In Europe, positive interpretation in IB testing is usually based on a single antigen, the OspC. We identified IgM antibodies to OspC in each of the 59 selected sera of the study patients.

Anti-*Borrelia* IgM may persist for years regardless of the history of a symptomatic infection (9–11). Because the etiology of this phenomenon has not yet been fully investigated, we studied its clinical relevance and the molecular background. The study participants were followed for a median of 6.2 months to track the natural course of IgM ELISA values. This time interval appeared sufficiently long to exclude patients with acute borrelial infection, as seroconversion to IgG would occur.

First, we investigated the relation of IgM levels to previous infection with *Borrelia*. Statistically significant differences in the values for OspCBsp and p 41 were observed in the IgM IB (Table 1). The reactivity was stronger in the study group. Likewise, the mean ELISA values were stronger in the study group compared to EM samples. A possible explanation could be that the immune response in EM patients might be in an early phase and the respective IgM values would increase in the course of the infection. Further, the IgM ELISA values depended on the time elapsed since past EM, becoming gradually weaker during the years after infection (Fig. 5). When focusing on the epitopes recognized by the persistent IgM antibodies, we concentrated on the 10 amino acid C-terminus of OspC that had previously been identified as a target sequence of IgM antibodies in *Borrelia*-infected patients (12). Using the OspC sequence of B. afzelii for epitope mapping, we found that the minimal epitope is the tetramer “PKKP,” which must be located at the C-terminus in order to be recognized by the persistent IgM antibodies. All tested patients recognized this minimal epitope, and some even recognized the trimeric sequence “PKK” or “KKP”. To investigate whether cross-reactions to this minimal epitope could occur, we performed a BLAST search for proteins with a C-terminal “PKKP” motif and found 2638 hits including two human proteins, human tryptase (TPSAB1) and synaptojanin-2 isoform X5. As surrogates of a potential cross-reactive autoantigen and a cross-reactive pathogen, we recombinantly expressed human TPSAB1 and P. aeruginosa Mg-chelatase with and without the C-terminal “PKKP,” respectively. Strikingly, the sera of patients with persistent IgM recognized both wild-type proteins but not when PKKP was deleted. Thus, it is tempting to speculate that IgM persistence in LB potentially originates from a previous infection with Lyme borreliae but is kept up in some individuals by continuous stimulation with cross-reactive autoantigens or antigens from other microorganisms or environmental factors. In respect to the latter, components of our diet could also contribute to the phenomenon of IgM persistence. For example, glutathioine transferase of important crops, such as common wheat (Triticum aestivum) and barley (*Hordeum vulgare*) also feature a C-terminal “PKKP”. Finally, polyclonal stimulation of B cells by nonrelated agents could also elicit IgM antibodies against *Borrelia*, as described for *Parvovirus B19* (7).

A source of the persistent IgM antibodies could be innate-like B cells that produce natural antibodies independently of T cell help. These IgM antibodies are often self- and/or polyreactive, thus recognizing conserved epitopes that are shared by foreign and autologous antigens. In this way, these natural antibodies facilitate both rapid neutralization of invading pathogens and promotion of “housekeeping” functions such as clearance of apoptotic cells (14). Indeed, in an earlier study, it was demonstrated that Borrelia IgM can arise from a T cell-independent B cell response in a mouse model (15).

In 44 samples, IgM reactivity to *Borrelia* proteins other than OspC was found by IB in our study. However, we selected OspC for the cross-reactivity studies because this protein plays a major role in the serological diagnosis of LB in many commercial ELISA and IB assays in Europe. A test result is considered positive if reactivity to OspC is detected. Therefore, investigating possible cross-reactions of this protein is crucial for the improvement of serological tests for LB.

It is highly controversial whether infection with B. burgdoferi sl can cause nonspecific complaints without other typical symptoms of LB. Seroprevalence to B. burgdoferi sl was shown to have no effect on such symptoms (16). When such symptoms occur after an episode of LB which was treated according to recommendations, the term *post-Lyme disease syndrome* (PLDS) has been suggested (17). However, sound evidence that such complaints are significantly more frequent after *Borrelia* infection is missing (18). In our study, we did not find an association between the ELISA value and number of symptoms. Our experimental results suggest that persistent IgM may be triggered by infection with Lyme *Borrelia* followed by maintenance through *Borrelia-*unrelated antigens. We speculate that autoimmunity may be associated with nonspecific symptoms. This assumption is supported by the IgM ELISA values, which were significantly higher in patients who reported no improvement (Fig. 7). Noteworthy, participants were not aware of the result of their follow-up serological tests and their assessment was thus not influenced by their knowledge about the serological status. These findings are in line with other studies investigating possible mechanisms for subjective disorders in PLDS patients. For instance, persistent symptoms may be associated with the immune response of the host, as demonstrated in an investigation of certain antibody profiles (19), and elevation of certain cytokines (20). In this study, we could not address all possible reasons for IgM persistence. However, its triggering by the C-terminal “PKKP” motif of Borrelia OspC followed by maintenance through the same motif on autoantigens and/or environmental antigens is an attractive explanation for PLDS and the basis for further studies.

Possible limitations of the study may be because nonspecific complaints were based on participants’ reports, the limited sample size and the lack of a healthy control group.

## MATERIALS AND METHODS

The study was approved by the Ethics Committee of the Medical University of Vienna (No. 1864/2012). The participants gave their written consent.

### Study population.

The participants were enrolled at the outpatient clinic of the Institute for Hygiene and Applied Immunology of the Medical University of Vienna. They were referred by physicians or self-referred mainly because of unspecific complaints and/or *Borrelia*-specific IgM antibody reactivity. The main inclusion criterion for participation in the study was a positive *Borrelia* IgM and a negative *Borrelia* IgG tested by ELISA and IB at three independent time points. Participants were enrolled between January 2012 and December 2014. None of the participants had symptoms of LB according to published clinical case definitions (2). Sixty-five participants were enrolled in the study and six of them were lost to follow-up. Thus, the study group included 59 participants of whom 13 (22%) were male and 46 (78%) were female. The mean age was 52 years (range 19–73 years). The sera from 14 patients with EM who had visited the outpatient department of the Institute and had been tested positive for anti-*Borrelia* IgM were used as a control group. Blood was drawn before antibiotic treatment. Four (28%) of these participants were male and 10 (72%) female, with a mean age of 46 years (range 30–71 years).

All study participants were seen by the first author of the study. At the initial visit, the participants were asked about the history and exact date of physician-diagnosed EM. The nonspecific complaints were documented with a questionnaire and included joint pain, joint swelling, back pain, muscle pain or muscle cramp, fatigue, forgetfulness, headache, visual disorders, vertigo, and sensory disorders. The participants were also asked about previous antibiotic treatment for IgM persistence. Follow-up visits were scheduled 4 weeks and 6 months after the initial visit. At the 6-month follow-up visit, the participants were asked whether their symptoms had improved or deteriorated compared to their physical condition at study inclusion.

### Serological testing.

The Borrelia ELISA manufactured by medac (Wedel, Germany) is based on *Borrelia*-specific VlsE peptide and outer surface protein (Osp) C antigen. The persistence of IgM was defined by a repeated positive test result at the 4-week follow-up visit and the 6-month follow-up visit. All results identified as >200 AU/ml were presented as 200 AU/ml. For inclusion in the study, a negative IgG ELISA was required at all three time points. Each patient was also tested by IB (Euroline-RN-AT, Euroimmun, Lübeck, Germany). IgM reactions to the following antigens were measured by automated, photometric reading of the visualized nitrocellulose membranes: VlsE, p25 (OspC), p39 (Borrelia membrane protein A), and p41. Presence of OspC, VlsE, or p39 band, either alone or in combination, was considered as a positive IB result.

The presence of cross-reactive viral antibodies was tested by IB (Euroimmun, Lübeck, Germany) according to the manufacturer’s instructions. The investigation focused on HSV, CMV, EBV, and P19V.

Patients with current EM were tested by ELISA and IB for IgG and IgM against Borrelia burgdorferi sl. Their IgM-positive sera (*n* = 14) were used as controls for the study group with persistent IgM antibodies. 10 samples were also IgG positive.

### Reactivity to OspC epitopes.

For cloning, E. coli host strains TOP10F´ (Thermo Fisher Scientific, Vienna, Austria) or NEB 5-alpha (New England Biolabs, Frankfurt am Main, Germany) were used and cultivated in lysogeny broth medium at 37°C. The primer sequences used for cloning are listed in Table S1 in the supplemental material. Recombinant full-length OspC and mutant variants (Table 3) of a previously identified C-terminal IgM epitope were constructed (12). Initially, the gene for OspC was PCR-amplified from B. afzelii strain PKO using primers BaPKO_OspC_F and BAPKO_OspC_R. For generation of variant 0, a version lacking the 20 C-terminal amino acids, primer BAPKO_OspC_REV_deltaC was used. The PCR products were cloned into expression vector pRSET-C (Thermo Fisher Scientific, Vienna, Austria) giving rise to vectors pRSET-C-bapko-OspC or pRSET-C-bapko-variant 0. Correct insertion of the respective amplicons was confirmed by sequencing (Microsynth, Vienna, Austria). The resulting vector pRSET-C-bapko-OspC was used as a template for site-directed mutagenesis (sdm) using the Q5 site-directed mutagenesis kit (New England Biolabs, Frankfurt am Main, Germany) as instructed by the manufacturer. The primers for sdm were designed with NEBase Changer v.1.2.6. (New England Biolabs, http://nebasechanger.neb.com/) and are listed in Table S2. The clones were screened for the correct mutation by sequencing (Microsynth, Vienna, Austria). For expression cloning of recombinant human tryptase 1 (TPSAB1), the myeloid cell line THP-1 was cultivated as previously described (21). Subsequently, RNA was isolated from these cells using the RNeasy kit (Qiagen, Hilden, Germany). The obtained RNA was then used to generate cDNA using the SSo Advanced kit (Bio-Rad, Vienna, Austria). The cloning fragment was generated by PCR using primers TPSAB1_Fwd and TPSAB1_Rev. The resulting fragment was cloned into expression vector pRSET-A (Thermo Fisher Scientific, Vienna, Austria) giving rise to plasmid pRSET-A_TPSAB1. Correct insertion of the fragment into the vector was confirmed by sequencing (Microsynth, Vienna, Austria). Deletion of the C-terminal “PKKP” motif was done with the Q5 site-directed mutagenesis kit (New England Biolabs, Frankfurt am Main Germany) using primers TPSAB1_ΔPKKP_fwd and TPSAB1_ΔPKKP_rev.

For cloning of P. aeruginosa Mg-chelatase P. aeruginosa magnesium chelatase (*bchI*), in-house strain P. aeruginosa 26071 was used. After the initial isolation, this strain was cultivated in brain heart infusion medium at 37°C. Before cloning of *bchI*, the presence of the C-terminal “PKKP” was confirmed by sequencing using primers P_aeruginosa_mg_seq_fwd and P_aeruginosa_mg_seq_rev. Subsequently, *bchI* was cloned into pRSET-A as described above, using primers Mg-Chelatase_fwd and Mg-Chelatase_rev. Deletion of PKKP was achieved using primers MgCh_ΔPKKP_fwd and MgCh_ΔPKKP_rev in combination with the Q5 site-directed mutagenesis kit (New England Biolabs, Frankfurt am Main, Germany) as above.

For protein expression from the cloned genes, the inserts were sub-cloned into E. coli Bl21(DE3)pLysS (Thermo Fisher Scientific, Vienna, Austria) or Rosetta 2 (Merck, Darmstadt, Germany). After expression, the six histidine (His-) tagged proteins were purified by gravity flow chromatography using Ni-NTA agarose (Qiagen, Hilden, Germany).

Purified proteins were separated by SDS-PAGE and blotted on a nitrocellulose membrane, which was blocked with Tris-buffered saline-Tween 20 solution (TBS-T; 25 mM Tris base, 137 mM NaCl, 2.7 mM KCl, 0.05% Tween 20, pH 8.0) containing 5% wt/vol milk powder. Visualization of successful expression of the recombinant proteins was confirmed using a His-probe antibody (sc-8036, Santa Cruz Biotechnology, Heidelberg, Germany). The membrane was developed using Pierce ECL Western Blotting Substrate (Thermo Fisher Scientific, Vienna, Austria). For testing patient sera, the respective serum was diluted 1:100 in TBS-T and the membranes were incubated for 1 h at room temperature. After thorough washing, the membrane was incubated for 1 h with an anti-human-IgM antibody conjugated with alkaline phosphatase (Euroimmun, Lübeck, Germany). The bands were visualized with 1-Step NBT/BCIP Substrate Solution (Thermo Fisher Scientific, Vienna, Austria).

### Statistical analysis.

Differences in IB band intensities between the study group and EM patients were assessed by two-sample t-tests. To correct for multiple testing, the *P values* were adjusted according to a resampling-based max-T procedure.

To analyze the association of EM and IgM development in the study group, a linear mixed model was calculated with ELISA as the dependent variable and history (yes or no), time during follow-up, and the interaction between the latter two as independent variables.

An exponential regression model was estimated to describe the nonlinear association between ELISA and the time since last EM (in years).

The relation between the ELISA value at the first visit and the number of nonspecific symptoms was calculated using one-way ANOVA.

A linear mixed model was estimated modeling ELISA as a function of the independent variables improvement of symptoms (yes or no), time (in months), and the interaction between time and improvement. A random intercept term for patients was included in both mixed models to account for the correlation of repeated measurements from the same patient.

The significance level for all tests was set to 5%. The software package R 3.3.0 was used for all analyses (22). The multtest package was used for the max-T procedure and the nlme package for the linear mixed model (23, 24).

## References

[1] Stanek G, Wormser GP, Gray J, Strle F. 2012. Lyme borreliosis. Lancet 379:461–473. doi:10.1016/S0140-6736(11)60103-7. 21903253

[2] Stanek G, Fingerle V, Hunfeld K-P, Jaulhac B, Kaiser R, Krause A, Kristoferitsch W, O'Connell S, Ornstein K, Strle F, Gray J. 2011. Lyme borreliosis: Clinical case definitions for diagnosis and management in Europe. Clin Microbiol Infect 17:69–79. doi:10.1111/j.1469-0691.2010.03175.x. 20132258

[3] Aguero-Rosenfeld ME, Wang G, Schwartz I, Wormser GP. 2005. Diagnosis of Lyme borreliosis. Clin Microbiol Rev 18:484–509. doi:10.1128/CMR.18.3.484-509.2005. 16020686 PMC1195970

[4] Fung BP, McHugh GL, Leong JM, Steere AC. 1994. Humoral immune response to outer surface protein C of *Borrelia burgdorferi* in Lyme disease: role of the immunoglobulin M response in the serodiagnosis of early infection. Infect Immun 62:3213–3221. doi:10.1128/iai.62.8.3213-3221.1994. 8039891 PMC302948

[5] Markowicz M, Kivaranovic D, Stanek G. 2015. Testing patients with non-specific symptoms for antibodies against Borrelia burgdorferi sensu lato does not provide useful clinical information about their aetiology. Clin Microbiol Infect 21:1098–1103. doi:10.1016/j.cmi.2015.08.005. 26321669

[6] Goossens HA, Nohlmans MK, van den Bogaard AE. 1999. Epstein-Barr virus and cytomegalovirus infections cause false-positive results in IgM two-test protocol for early Lyme borreliosis. Infection 27:231. doi:10.1007/BF02561539. 10378140

[7] Tuuminen T, Hedman K, Söderlund-Venermo M, Seppälä I. 2011. Acute parvovirus B19 infection causes nonspecificity frequently in *Borrelia* and less often in *Salmonella* and *Campylobacter* serology, posing a problem in diagnosis of infectious arthropathy. Clin Vaccine Immunol 18:167–172. doi:10.1128/CVI.00367-10. 21106777 PMC3019781

[8] Hillerdal H, Henningsson AJ. 2021. Serodiagnosis of Lyme borreliosis-is IgM in serum more harmful than helpful? 2021. Eur J Clin Microbiol Infect Dis 40:1161–1168. doi:10.1007/s10096-020-04093-2. 33409833 PMC8139919

[9] Kalish RA, McHugh G, Granquist J, Shea B, Ruthazer R, Steere AC. 2001. Persistence of immunoglobulin M or immunoglobulin G antibody responses to *Borrelia burgdorferi* 10–20 years after active Lyme disease. Clin Infect Dis 33:780–785. doi:10.1086/322669. 11512082

[10] Lantos PM, Lipsett SC, Nigrovic LE. 2016. False positive Lyme disease IgM immunoblots in children. J Pediatr 174:267–269. doi:10.1016/j.jpeds.2016.04.004. 27157898 PMC4925275

[11] Seriburi V, Ndukwe N, Chang Z, Cox ME, Wormser GP. 2012. High frequency of false positive IgM immunoblots for *Borrelia burgdorferi* in clinical practice. Clin Microbiol Infect 18:1236–1240. doi:10.1111/j.1469-0691.2011.03749.x. 22369185

[12] Mathiesen MJ, Holm A, Christiansen M, Blom J, Hansen K, Ostergaard S, Theisen M. 1998. The dominant epitope of *Borrelia garinii* outer surface protein C recognized by sera from patients with neuroborreliosis has a surface-exposed conserved structural motif. Infect Immun 66:4073–4079. doi:10.1128/IAI.66.9.4073-4079.1998. 9712750 PMC108488

[13] Wormser GP, Tang AT, Schimmoeller NR, Bittker S, Cooper D, Visintainer P, Aguero-Rosenfeld ME, Ogrinc K, Strle F, Stanek G. 2014. Utility of serodiagnostics designed for use in the United States for detection of Lyme borreliosis acquired in Europe and vice versa. Med Microbiol Immunol 203:65–71. doi:10.1007/s00430-013-0315-0. 24218117

[14] Palm AE, Kleinau S. 2021. Marginal zone B cells: From housekeeping function to autoimmunity? J Autoimmun 119:102627. doi:10.1016/j.jaut.2021.102627. 33640662

[15] Tunev SS, Hastey CJ, Hodzic E, Feng S, Barthold SW, Baumgarth N. 2011. Lymphoadenopathy during lyme borreliosis is caused by spirochete migration-induced specific B cell activation. PLoS Pathog 7:e1002066. doi:10.1371/journal.ppat.1002066. 21637808 PMC3102705

[16] Hjetland R, Reiso H, Ihlebæk C, Nilsen RM, Grude N, Ulvestad E. 2015. Subjective health complaints are not associated with tick bites or antibodies to Borrelia burgdorferi sensu lato in blood donors in western Norway: a cross-sectional study. BMC Public Health 15:657. doi:10.1186/s12889-015-2026-5. 26169496 PMC4499943

[17] Wormser GP, Dattwyler RJ, Shapiro ED, Halperin JJ, Steere AC, Klempner MS, Krause PJ, Bakken JS, Strle F, Stanek G, Bockenstedt L, Fish D, Dumler JS, Nadelman RB. 2006. The clinical assessment, treatment, and prevention of Lyme disease, human granulocytic anaplasmosis, and babesiosis: clinical practice guidelines by the Infectious Diseases Society of America. Clin Infect Dis 43:1089–1134. doi:10.1086/508667. 17029130

[18] Obel N, Dessau RB, Krogfelt KA, Bodilsen J, Andersen NS, Møller JK, Roed C, Omland LH, Christiansen CB, Ellermann-Eriksen S, Bangsborg JM, Hansen K, Benfield TL, Rothman KJ, Sørensen HT, Andersen CØ, Lebech AM. 2018. Long term survival, health, social functioning, and education in patients with European Lyme neuroborreliosis: nationwide population based cohort study. BMJ 361:k1998. doi:10.1136/bmj.k1998. 29848547 PMC5974636

[19] Chandra A, Wormser GP, Marques AR, Latov N, Alaedini A. 2011. Anti-*Borrelia burgdorferi* antibody profile in post-Lyme disease syndrome. Clin Vaccine Immunol 18:767–771. doi:10.1128/CVI.00002-11. 21411605 PMC3122515

[20] Strle K, Stupica D, Drouin EE, Steere AC, Strle F. 2014. Elevated levels of IL-23 in a subset of patients with post-lyme disease symptoms following erythema migrans. Clin Infect Dis 58:372–380. doi:10.1093/cid/cit735. 24218102 PMC3890340

[21] Machacek C, Supper V, Leksa V, Mitulovic G, Spittler A, Drbal K, Suchanek M, Ohradanova-Repic A, Stockinger H. 2016. Folate receptor β regulates integrin CD11b/CD18 adhesion of a macrophage subset to collagen. J Immunol 197:2229–2238. doi:10.4049/jimmunol.1501878. 27534550

[22] R Core Team. 2016. R: A language and environment for statistical computing. R Foundation for Statistical Computing, Vienna, Austria. https://www.R-project.org/.

[23] Pollard KS, Dudoit S, van der Laan MJ. 2005. Multiple testing procedures: R multitest package and applications to genomics, p 251–272. *In* Gentleman R, Carey VJ, Huber W, Irizzarry R, Dztoit S (ed), Bioinformatics and computational biology solutions using R and bioconductor. Springer, New York, NY.

[24] Pinheiro J, Bates D, DebRoy S, Sarkar D, R Core Team. 2016. nlme: Linear and nonlinear mixed effects models (Software). R package version 3.1–125. http://CRAN.R-project.org/package=nlme.

