# Supplementary material for: Persistent Anti-Borrelia IgM Antibodies without Lyme Borreliosis in the Clinical and Immunological Context
Source: Microbiol Spectr. 2021 Dec 22;9(3):e01020-21. doi: 10.1128/Spectrum.01020-21 (PMC8694107; doi:10.1128/Spectrum.01020-21)
Supplement: SUPPLEMENTAL FILE 2 — Supplemental material. Download SPECTRUM01020-21_Supp_1_seq7.pdf, PDF file, 0.5 MB [file spectrum01020-21_supp_1_seq7.pdf]

Supplemental Table 1. Primers used for cloning of recombinant proteins. Cutting sites of restriction enzymes are underscored.

**fusion protein**	**primer designation**	**primer sequence**
OspC	BaPKO_ospC_F	5'-GGTGGTGGATCCATAATTCAGGGAAAGGTGGG-3'
	BAPKO_ospC_R	5'-GGTGGTCTCGAGTGACTTTATTTTTCCAGTTACTT-3'
human tryptase 1 (TPSAB1)	TPSAB1_fwd	5´-GATGATCTCGAGCTGAATCTGCTGCTGCTGG-3´
	TPSAB1_rev	5´-GATGATGAATTCTTACGGCTTTTTGGGGACATAGT-3`
human tryptase 1_ΔPKKP	TPSAB1_ΔPKKP_fwd	5'-TAAGAATTCGAAGCTTGATCC-3'
	TPSAB1_ΔPKKP_rev	5'-GACATAGTGGTGGATCCAG-3'
P. aeruginosa Mg-chelatase (*bchI)*	P_aeruginosa_mg_seq_fwd	5'-GAACATTTCGCCCTGCTGCAT-3'
	P_aeruginosa_mg_seq_rev	5'-ATCCGCACCCATGACCG-3'
	Mg-Chelatase_fwd	5'- TAATAACTCGAGAGCGCTACCGTGCATTAC-3'
	Mg-Chelatase_rev	5'- ATACTAGAATTCTTAGGGCTTTTTTGGCC-3'
P. aeruginosa Mg-chelataseΔPKKP (*bchI)*	MgCh_ΔPKKP_fwd	5´TAAGAATTCGAAGCTTGATCCGGCTGCTAACAAAGC-3´
	MgCh_ΔPKKP_rev	5´-CCAGCGCGGCAGCTCGCG-3´

Supplemental Table 2. OspC variants 0, and A-M created from the full-length protein. For variant 0 the amplicon was cloned into vector pRSET-C (Thermo Fisher Scientific, Austria). The cutting sites of the restriction enzymes used are underscored. Other variants were generated by site directed mutagenesis (sdm) using the primers listed. For variants A-G and L-M, vector pRSET-C-bapko-ospC was used as a PCR template for sdm and reverse primer 5’-ACTTGTAAGTTCTTTAACTGAATTAG-3’ was used together with the listed primer sequences. For variant H forward and reverse primers are listed and pRSET-C-bapko-ospC was used as a PCR template. For variant I forward and reverse primers are listed and variant A was used as a template for amplification. For variants J-K forward primer 5´-TAATTAGATCAATATTATAAGATTAATTTG-3´ was used together with the listed reverse primers and pRSET-C-bapko-ospC was used as a template for sdm PCR.

OspC **variant**	**primer sequence(s)**
0	5’-GGTGGT<u>GGATCC</u>ATAATTCAGGGAAAGGTGGG-3’ (BaPKO_ospC_F)<br>5’-GGTGGT<u>CTCGAG</u>GTGCTACTTGAGCTGCTTTTA-3’ (BAPKO_ospC_REV_deltaC)
A	5‘-TAATTAGATCAATATTATAAGATTAATTTG-3‘
B	5‘-CCAAAAAAACCTTAATTAGATC-3‘
C	5‘-AGTCCAAAAAAACCTTAATTAG-3‘
D	5‘-GAAAGTCCAAAAAAACCTTAATTAG-3‘
E	5‘-GCAGAAAGTCCAAAAAAAC-3‘
F	5‘-GTAGCAGAAAGTCCAAAAAAAC-3‘
G	5‘-GTTGTAGCAGAAAGTCCAAAAAAAC-3‘
H	5’-TAATTAGATCAATATTATAAGATTAATTTG-3‘<br>5‘-ACTTTCTGCTACAACAGG-3‘
I	5’-AGTCCAAAAAAACCTGAATTAAAGACAGAAATTGCAAAG-3’<br>5’-TTCTGCTACAACAGGTTCTAAATTTTTCAATTTACTCAATTTTTC-3’
J	5´-TTTTTTTGGACTTTCTGCTAC-3´
K	5´-TTTTGGACTTTCTGCTAC-3´
L	5´-AAAAAACCTTAATTAGATCAATATTATAAG-3´
M	5´-AAACCTTAATTAGATCAATATTATAAG-3´
